# Recent Development of Advanced Fluorescent Molecular Probes for Organelle-Targeted Cell Imaging

**DOI:** 10.3390/bios13030360

**Published:** 2023-03-08

**Authors:** Sha Lu, Zhiqi Dai, Yunxi Cui, De-Ming Kong

**Affiliations:** 1 State Key Laboratory of Medicinal Chemical Biology, Tianjin Key Laboratory of Biosensing and Molecular Recognition, Research Centre for Analytical Sciences, College of Chemistry, Nankai University, Tianjin 300071, China; 2 College of Life Sciences, Nankai University, Tianjin 300071, China

**Keywords:** cell imaging, organelle targeting, fluorescence, molecular probes

## Abstract

Fluorescent molecular probes are very powerful tools that have been generally applied in cell imaging in the research fields of biology, pathology, pharmacology, biochemistry, and medical science. In the last couple of decades, numerous molecular probes endowed with high specificity to particular organelles have been designed to illustrate intracellular images in more detail at the subcellular level. Nowadays, the development of cell biology has enabled the investigation process to go deeply into cells, even at the molecular level. Therefore, probes that can sketch a particular organelle’s location while responding to certain parameters to evaluate intracellular bioprocesses are under urgent demand. It is significant to understand the basic ideas of organelle properties, as well as the vital substances related to each unique organelle, for the design of probes with high specificity and efficiency. In this review, we summarize representative multifunctional fluorescent molecular probes developed in the last decade. We focus on probes that can specially target nuclei, mitochondria, endoplasmic reticulums, and lysosomes. In each section, we first briefly introduce the significance and properties of different organelles. We then discuss how probes are designed to make them highly organelle-specific. Finally, we also consider how probes are constructed to endow them with additional functions to recognize particular physical/chemical signals of targeted organelles. Moreover, a perspective on the challenges in future applications of highly specific molecular probes in cell imaging is also proposed. We hope that this review can provide researchers with additional conceptual information about developing probes for cell imaging, assisting scientists interested in molecular biology, cell biology, and biochemistry to accelerate their scientific studies.

## 1. Introduction

The cell is the fundamental constituent of living bodies and one of the basic units for biological investigation. At the subcellular level, various organelles, such as nuclei, mitochondria, endoplasmic reticulums (ERs), lysosomes, etc., are distributed inside cells and function synergistically. Thus far, the statuses of different organelles during distinct bioprocesses have attracted intense interest. It is important to provide visualized organelle signals in biological studies because the relative localizations between different organelles or between organelles and particular molecules significantly influence their activations. For example, the release of cytochrome C from mitochondria into the cytoplasm is a special marker of apoptosis [1,2,3]. A large number of proteins, especially transcription factors (TFs), may transfer from other locations into the nucleus to initiate downstream gene expression under different cell stress conditions, such as heat shock [4] and ER stress [5,6,7]. Mitochondria–ER contact (MERC) is important for intracellular calcium homeostasis and other metabolic processes [8,9]. Immunofluorescence (IF) is a widely used method for staining organelles with high specificity [10]. Usually, a primary antibody of a unique biomarker molecule of the special organelle is first incubated with cells to recognize and bind to the target. A secondary antibody labeled with a fluorophore will then be applied to bind to the primary antibody and give the signal. This IF method is highly specific but has some limitations. The recognition between the primary and secondary antibodies relies on the resource species of the primary antibody. For reasons of costs, limited resource species, such as mice, rabbits, and goats, have been chosen, such that the IF method can provide only a few different signals at one time. More importantly, the cells used for IF staining must be fixed and permeabilized to allow the large antibody molecules to enter the targeted localization, such that it cannot be applied to the real-time monitoring of the statuses of organelles in living systems. Due to the development of molecular biology and the discovery of fluorescence proteins (FPs), researchers in the field of biology can construct artificial proteins that fuse FPs with special signal peptides to endow FPs with the capacity of organelle targeting [11]. This method lightens particular organelles and can work in living cells with great specificity; however, the fused proteins have to be expressed intracellularly through elaborate experimental operations involving toxic cell transfection reagents. In addition, the exogenous protein may cause some unexpected bioprocess, such as immune responses [12].

Molecular probes with high specificity toward particular organelles are promising tools for biological studies at the subcellular level [13]. Through the contemporary development of molecular biology, more knowledge can be gained about the particular characteristics of organelles, such as pH and potential. Some proteins have also been found to have unique localizations and may be marked by unique molecules. Hence, scientists can design probes based on these special senses. Some commercial tracker probes have already been employed to stain special organelles, such as nuclei [14], mitochondria [15], ERs [16], and lysosomes [17]. With a particular design, small fluorescent molecules can penetrate the membranes of living cells and target particular organelles with high efficiency.

Up until now, the understanding of cells has extended to the molecular level, where the focus is on more detailed parameters inside cells and even inside unique organelles [18]. For example, scientists have indicated that the pH inside the lysosome is much lower than the normal physiological pH of 7.4 [19], that the temperature of the mitochondrial matrix may be as high as 50 °C [20], that the calcium transient on ER surfaces initiates autophagy [21], and that increased concentrations of Fe^2+^ ions may cause a new kind of programmable cell death process called ferroptosis [22]. These detailed molecular features have attracted more and more attention because they have been proven to be closely related to numerous bioprocesses and diseases. Therefore, a clear understanding of molecular characteristics at the subcellular level will help explain molecular mechanisms in biology and pathology, thus accelerating studies on the diagnosis and therapeutics of diseases, such as cancers, cardiovascular diseases, and even the recent SARS-CoV-2. To achieve a precise evaluation of the intracellular molecular substances at the organelle level, it is important to develop novel molecular probes that can simultaneously target particular organelles and respond to distinct parameters. The most popular method for designing specific probes is linking different functional molecules as a whole to construct a new molecule with multiple functions. The key to this method is to find and develop appropriate functional groups and linkers to ensure that they can keep their original properties. In this review, we will introduce the developed molecular probes that have emerged in the last decade, where the probes have been applied in the imaging of unique parameters at the organelle level. We will discuss the crucial organelles, including nuclei, mitochondria, ERs, and lysosomes, and introduce the general strategy in designing molecules for specific targeting. Moreover, we will also discuss factors related to different organelles, such as the concentrations of ions as well as bioactive molecules, and the physical properties of the special organelles. We will discuss the meaning of each parameter and the strategies used to monitor them. We hope that this general review can provide some new opinions for people interested in both the chemical and biological fields, to dig out promising molecular probes to better explain the mechanism of different bioprocesses to provide advanced approaches for disease diagnosis and treatment.

## 2. Design of Molecular Probes for Organelle-Targeted Cell Imaging

### 2.1. Nucleus-Targeted Molecular Probes

#### 2.1.1. Properties of the Nucleus

The cellular nucleus is the most important organelle inside a cell. The nucleus is the habitat of chromosomes—the fundamental components that store genetic information [23]. Chromosomes constitute DNA molecules that encode the gene information in their sequences, thus controlling the expression of proteins in cells. It is well-known that gene mutations will induce abnormal activity of proteins and influence the health of cells. Plenty of diseases have been proven to be related to the nucleus [24,25,26]. In addition, the status of the nucleus has been recognized as a biomarker of some cell-fate-related bioprocesses, such as apoptosis and necrosis [27,28].

The structure of the nucleus contains double-layer membranes enclosing the contents, which include the nucleolus, DNA, and other genetic materials. The outer nucleus membrane contains many structures termed nuclear pores, which regulate the exchange of molecules. Some small hydrophilic molecules can freely transfer across nuclear pores, whereas the macromolecules, such as nucleic acids and proteins, must pass the membrane with the facility of other transporters, called importins [29].

The abundance of DNA is the most distinguishing factor of the nucleus. Hence, many molecular probes have been designed to interact with DNA molecules for nucleus targeting. The most commonly utilized interaction methods between DNA and small molecules include electrostatic interaction, intercalation, and groove binding [30,31]. The phosphoric backbone of the long-chain DNA structure makes the whole molecule negatively charged, and thus can bind with cationic dyes through electrostatic force. Some molecules, such as anthracyclines and acridines, can intercalate in the double-stranded structure through interaction with the bases. Major and minor grooves are featured sites of the double-stranded DNA. Small molecules can bind along grooves through hydrogen binding interaction, especially narrower minor grooves. Moreover, the importin system was also utilized for nucleus targeting.

#### 2.1.2. Nucleus-Targeted Probe Design

**Scheme 1:**
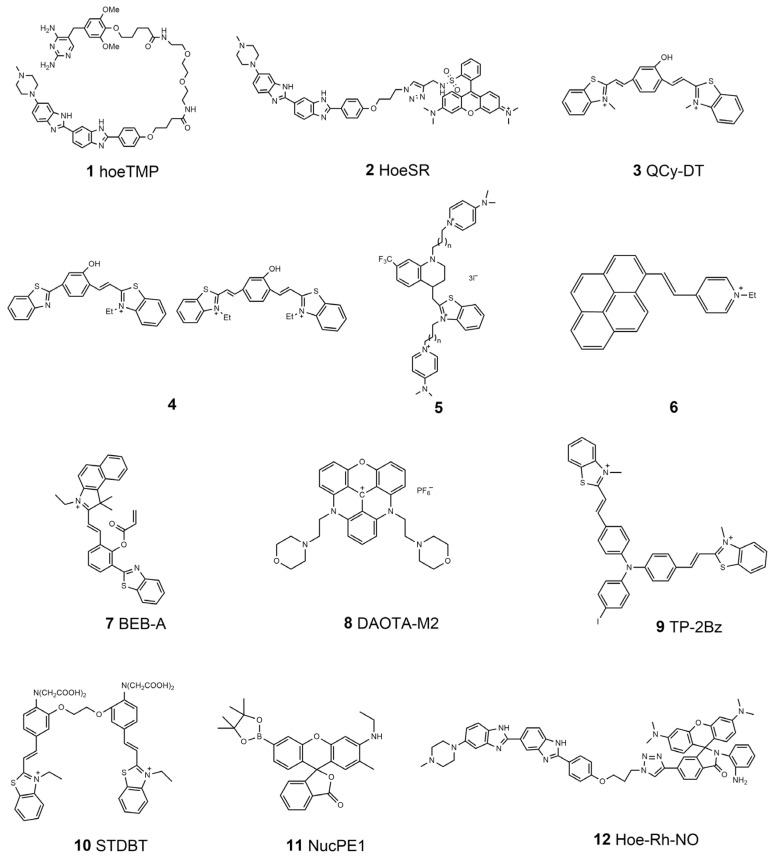
Chemical structures of nucleus-targeted fluorescent molecular probes.

As mentioned above, molecules that can accumulate around DNA are potential specific probes for nucleus targeting. At present, commercial dyes, such as DAPI, PicoGreen, and Hoechst series, have generally been applied to nucleus staining because they can bind with double-stranded DNA structures and display special fluorescent signals [32,33]. Hoechst is a typical molecule that can bind to DNA minor grooves at A–T-rich regions [34]. The basic structure of Hoechst has been used as the guide functional group with which to drive other fluorophores to nuclei. Hamachi’s group has introduced a new concept called self-localizing ligands (SLLs), which can bind with target proteins and tether them to a new position inside cells [35]. They demonstrated that a trimethoprim (TMP) links with Hoechst to form a new ligand (**hoeTMP**, **1**, Scheme 1) that can guide a special protein, dihydrofolate reductase (eDHFR), to nuclei with high efficiency. Inspired by this discovery, the same group developed a Hoechst tagging strategy that can simply guide non-specific molecular fluorophores towards the position of nuclei by linking them with Hoechst [36]. Recently, Zhang et al. reported a new derivative called **HoeSR** (**2**, Scheme 1), which was constructed by conjugating sulforhodamine (SR) and Hoechst, for the super-resolution imaging of nuclei in living cells through direct stochastic optical reconstruction microscopy (dSTORM) [37]. This HoeSR probe has been applied to chromosome morphology imaging at a super-resolution level, making the probe itself not only an indicator of nuclei but also a potential dye of DNA structures. In addition to the Hoechst moiety, some other unique chemical structures have also been reported to recognize DNA structures through groove binding. In 2015, Govindaraju and co-workers reported a DNA-specific probe, basing its design on the quinone cyanine-dithiazole (**QCy-DT**, **3**, Scheme 1) structures [38]. They demonstrated that **QCy-DT** could recognize duplex AT-rich DNA through the minor groove binding mechanism, which is the first switch-on NIR fluorescence probe for sequence-specific DNA staining. They also showed that the probe could be applied to specific parasite staining and treatment. Recently, they have explained the cis/trans isomerization of **QCy-DT** upon binding to DNA targets and promoted the application of the probe [39]. Pang’s group developed a novel NIR-emitting probe based on phenol-containing m-phenylene building blocks for DNA-targeted staining through a groove binding mechanism (**4**, Scheme 1). They showed that the probe modification on the m-phenylene bridge group might endow the probe with the ability to target other organelles, such as lysosomes in cells [40]. Intercalation is another general mode of interaction between small molecules and DNA [41,42], which led to the development of numerous molecular probes for DNA targeting and nucleus imaging based on the intercalation method. Acridine orange (AO) is a well-known commercial DNA-intercalating dye. The fluorescence property of AO has been explored further recently [43]. Kandinska et al. prepared a series of asymmetric monomeric monomethine cyanine dyes through a simple and reliable synthetic procedure (**5**, Scheme 1). They found that the designed probes can bind with DNA through either groove binding or intercalation processes. These probes showed excellent imaging properties of DNA and RNA, completely suitable for application as DNA and RNA fluorescent molecular probes [44]. Polycyclic aromatic hydrocarbons (PAHs), such as naphthalene, anthracene, pyrene, and perylene, are excellent rigid molecular skeletons that are useful for developing highly efficient two-photon excitable fluorescent probes. Ali et al. designed an innovative fluorescent dye by impregnating perylene diimide into the functionalized surface of magnetic core–shell silica nanoparticles [45]. These nanoparticles exhibit features such as low toxicity, being environmentally friendly, and high sensitivity; they also show high DNA binding capacity, which makes the developed particle promising for DNA extraction, delivery, and fluorescent labeling. Pang’s group synthesized a pyrene-based fluorescent probe (**6**, Scheme 1), which possesses remarkable selectivity to the intracellular nucleus and is also useful for two-photon fluorescence microscopy [42]. They proposed a possible mechanism: due to the rigid planar pyrene structure of it, a possible interaction mode is the intercalation of the molecules into adjacent base pairs in the DNA duplex. The nucleic RNA can also be a target for nucleus imaging. Zhao et al. developed a probe called **BEB-A** (**7**, Scheme 1) [46]. With the help of cysteine, the probe could bind with RNA molecules through the hydrogen bonds forming between the probe and the uracil in the RNA molecules, thus achieving accumulation in the nucleolus position. They showed that this **BEB-A** could image cysteine in mitochondria and RNA in the nucleus simultaneously.

The double helix is not the only existing structure of DNA molecules in living cells. For instance, guanine-rich DNA sequences were found to form a tetra-stranded structure called a G-quadruplex (GQ) [47,48]. Many studies have indicated that GQs are implicated in numerous bioprocesses, such as gene expression regulation and telomere replication [49,50]. Therefore, GQs provide a potential marker not only for nucleus-targeted imaging but also for gene activities. Most specific probes for the detection of GQs are based on the interaction between the probe molecules and the unique planar G-quartet units through π–π stacking. Vilar and co-workers designed a small molecule based on trianguleniums, termed **DAOTA-M2** (**8**, Scheme 1) [51]. They demonstrated that **DAOTA-M2** could recognize GQs and thus applied this probe to the identification of GQs in the nuclei of living cells using fluorescence lifetime imaging microscopy (FLIM) [52]. Sun et al. synthesized a new probe called **TP-2Bz** (**9**, Scheme 1) by linking triphenylamine with N-methylbenzothiazole [53]. **TP-2Bz** can bind with GQs for specific imaging and reveal the viscosity in the nucleus region because of the unique twistable donor–π–acceptor structure of the probe.

Signal transduction molecules within nuclei always serve important roles in regulating different bioactivities. Zhu et al. linked two benzothiazolium hemicyanine dyes with a special Ca^2+^-chelating molecule to construct a new probe, called **STDBT** (**10**, Scheme 1), for Ca^2+^ detection [54]. They found that the acetoxymethyl ester of **STDBT** distributes in both the cytosol and the nucleus with a clear boundary after entering cells; it could be applied to imaging nucleic Ca^2+^. Meanwhile, endogenous reactive species, such as H_2_O_2_ and NO, are general signaling molecules in the nucleus [55,56,57]. In 2011, Dickinson et al. designed the first probe, **NucPE1** (**11**, Scheme 1), for imaging nucleic H_2_O_2_ in living cells [58]. They demonstrated that this new probe works in both living cells and in vivo in *C. elegans*. They found that the nuclear ROS pools in *C. elegans* were regulated by a longevity-promoting sirtuin protein, demonstrating that NucPE1 is a potential tool for biological investigation. Recently, Zhao et al. developed a probe called **Hoe-Rh-NO** (**12**, Scheme 1) for the localized imaging of NO in nuclei [59]. They selected a rhodamine spirolactam as the NO indicator moiety and Hoechst as the nucleus guider. A summary of the properties of the nucleus-targeted molecular probes is listed in Table 1.

This probe was applied to the detection of both exo-/endogenous NO in a zebrafish inflammation model.

### 2.2. Mitochondria-Targeted Molecular Probes

#### 2.2.1. Property of the Mitochondria

Mitochondria, known as the “powerhouse” of cells, are important organelles that play a key role in sourcing energy for most creatures [60]. Mitochondria are the primary areas where adenosine triphosphate (ATP) is produced, which fuels most biological processes, such as cell proliferation, biogenesis, biosynthesis, and metabolism. During the development of modern biology, scientists found that the functions of mitochondria are far more complicated than simply that of a powerhouse. Mitochondria modulate the homeostasis of cellular Ca^2+^ and reactive oxygen species (ROS) [61,62]. They are also responsible for many cellular bioprocesses, such as autophagy and protein degradation [63,64]. In recent studies, mitochondria have been verified to regulate different programmed cell death processes, such as apoptosis, pyroptosis, and ferroptosis [65,66,67,68,69]. Because of the significance of mitochondria, it is not surprising that people find that they are closely related to numerous diseases, such as cancers, cardiovascular diseases (CVDs), and neurodegenerative disorders [70,71,72].

The basic structure of mitochondria contains a double lipid bilayer—the outer membrane and the inner membrane, the matrix coated by the inner membrane, and intermembrane space (IMS) [73]. The outer mitochondrial membrane (OMM) is permeable for molecules smaller than 6 kD, thus keeping the fundamental substance exchange between the cytoplasm and the IMS [74].

The oxidative phosphorylation and tricarboxylic acid cycle processes occur in the matrix to produce ATP through the synergistic function between proteins located in the matrix and the inner mitochondrial membrane (IMM). Notably, a proton transfer process occurs during these bioprocesses. The proton pump on the IMM may drive the protons from the matrix to the IMS, resulting in a membrane potential that is much higher than that of other organelles. In fact, the potential difference between the two sides of the IMM can be as large as 160–180 mV [75]. Therefore, molecules with a positive charge can be accumulated on the matrix side of the IMM spontaneously once they are able to permeate the lipid bilayer; this is the most general physical parameter of mitochondria for the principle of specific probe design.

#### 2.2.2. Mitochondria-Targeted Probe Design

**Scheme 2:**
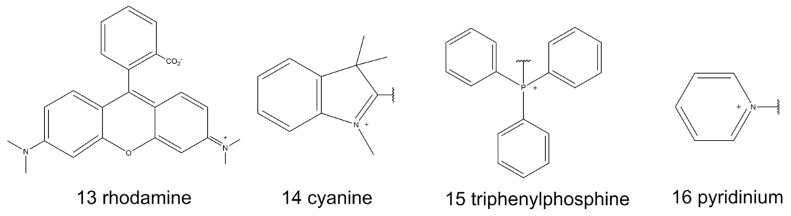
Chemical structures of typical mitochondria-targeted building blocks.

Thus far, the majority of the mitochondria-targeted molecular probes have been lipophilic cations, including intrinsic lipophilic cationic dyes, such as **rhodamine** (**13**, Scheme 2) and **cyanine** (**14**, Scheme 2). Moreover, **triphenylphosphine** (**TPP**, **15**, Scheme 2) and **pyridinium** (**16**, Scheme 2) are typical building blocks that can be modified as the mitochondria-targeted moieties on natural fluorophores [13,76]. The positive charge of the molecular probes is important for mitochondrial specificity because of the high potential of the IMM, as mentioned above.

The staining of mitochondria provides valuable insights into morphology. The word “mitochondrion” stems from Greek and contains two parts, “mitos” and “khondrion”, which mean thread and small grain, respectively. Natural mitochondria have a highly dynamic morphology, transitioning from dense branches to scattered particles through a fission–fusion process regulated by several proteins. At this time, commercial dyes, such as the MitoTracker series, are available for mitochondria-specific imaging. These powerful tools provide visualized signals of mitochondrial morphology and mitochondrial dynamic information to people for further studies on biology, pathology, and pharmacology [77]. Meanwhile, advanced molecular probes have been developed as structure probes of mitochondria with high specificity and low toxicity. Relying upon these developments, numerous probes have been applied to super-resolution cell imaging to help people in cellular and molecular biology monitor and investigate mitochondria-related bioprocesses [76].

**Scheme 3:**
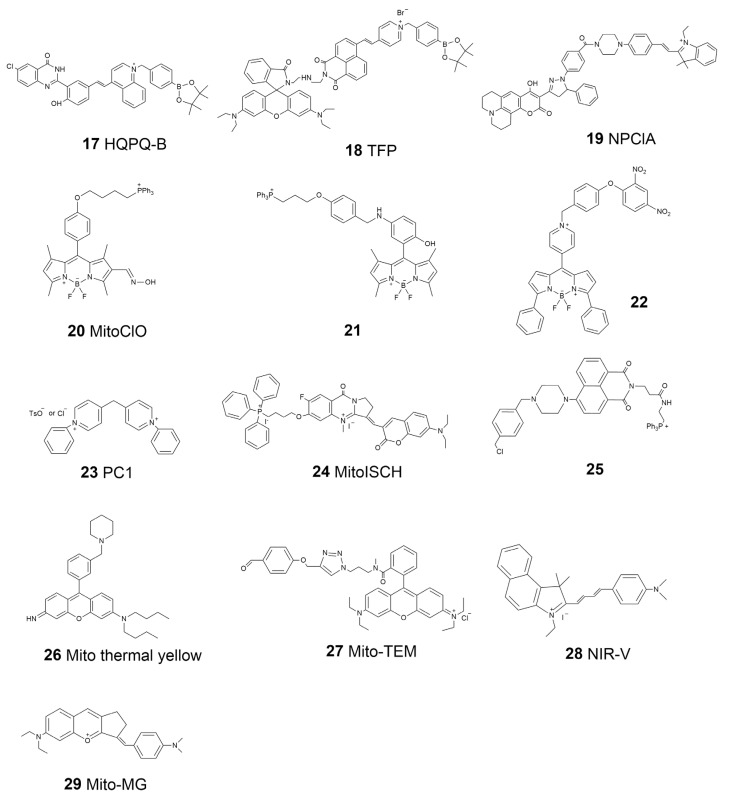
Chemical structures of mitochondria-targeted fluorescent molecular probes.

The structures and dynamics of mitochondria provide significant information for intracellular bioactivities. Moreover, mitochondria are the places that carry many important biochemical interactions inside cells. Therefore, probes that can investigate the functional properties of mitochondria are also important and have recently garnered increasing interest. For example, the membrane potential of mitochondria is vital to maintaining respiratory chain reactions, ion transfer, and other bioactivities. Several commercial probes, including TMRM and JC-1, have been widely applied to detect the mitochondria membrane potential [78,79]. Chemical reagents are also important for the normal activity of mitochondria. Mitochondria are the primary areas of ROS production. They produce ROS during the electron transfer mechanism in related bioprocesses, such as oxidative phosphorylation. ROS are important reagents in cells that play multiple roles, acting as signal transducers, stress inducers, and pathogen defenders [80]. A certain concentration of ROS is necessary for cells; however, the accumulation of ROS will cause oxidative damage to biomolecules such as DNA, proteins, and lipids [81]. MitoSOX is a commercial dye used to visualize the broad-spectrum ROS in mitochondria [82]. Thus far, multifunction probes with better performance have been introduced. Li et al. reported a probe, **HQPQ-B** (**17**, Scheme 3), for the highly accurate detection of mitochondrial H_2_O_2_ [83]. The probe would precipitate and show a signal in situ once it reacted with H_2_O_2_, thus avoiding the false signal caused by the dispersion of traditional soluble probes. Recently, Zhou et al. designed a two-photon fluorescence-lifetime-based probe (**TFP**, **18**, Scheme 3) that can monitor the ATP and H_2_O_2_ levels of mitochondria in living cells in real time [84]. **TFP** is a potential tool for the study of the relationship between cellular oxidative state and energy metabolism. Niu et al. developed a two-photon three-channel fluorescence probe, **NPClA** (**19**, Scheme 3), which responds to SO_2_ and ClO^−^ for more detailed mitochondrial oxidative stress imaging [85]. An oxime-containing fluorescent probe, **MitoClO** (**20**, Scheme 3), based on a BODIPY scaffold and the C=N isomerization mechanism, was designed by Peng’s group and used for HClO determination with a rapid response, low detection limits, and high selectivity. Triphenylphosphine was introduced at the meso-position to enable the probe to locate mitochondria within living cells [86].

ROS are not the only attractive compounds inside mitochondria. Because of the complex function of mitochondria, several metal ions and other reactive species, such as reactive nitrogen species (RNS) and reactive sulfur species (RSS), are involved in maintaining the activity of mitochondria [87,88]. Nowadays, probes for the mitochondria local imaging of ions such as Ca^2+^, Zn^2+^, Fe^2+^, Hg^2+^, RNS, such as NO, RSS, such as H_2_S, and glutathione (GSH) have been introduced; a variety of them are commercially available [89,90,91,92,93,94]. Based on the N-nitrosation of aromatic secondary amines, Miao et al. developed a mitochondrion-targeted NO probe (**21**, Scheme 3) and successfully visualized the NO levels in macrophages in the resting state or under diverse stimulations [95]. In this BODIPY-based probe, TPP was introduced as the targeting group, where a N-benzyl-4-hydroxyaniline moiety was the reaction site. This probe was applied to detect NO levels in living cells after oxygen–glucose deprivation (OGD) stimulation. Zhang et al. reported a BODIPY-based probe (**22**, Scheme 3) for monitoring mitochondrial GSH [96]. They successfully used this probe for mitochondrial-targeted GSH imaging in living HeLa cells. The self-immolative group paradinitrophenoxy benzyl pyridinium was introduced at the meso-position of BODIPY, and it could simultaneously act as the targeting ligand and responsive group.

Mitochondria are known as semi-autonomous organelles, which means that they have their own genetic system, including mitochondrial DNA (mtDNA) and related enzymes for gene transcription [97]. Human mtDNA is a closed-circle model constructed by double-stranded DNA with a length of 16,569 base pairs. Traditional biology has revealed that mtDNA encodes a few RNAs and peptides that function for mitochondria. Moreover, an increasing number of studies have indicated that mtDNA is also related to different physiological processes, such as aging and inflammation [98]. Therefore, the imaging of mtDNA has become interesting in recent years. The most challenging part of mtDNA staining is the interference from nuclear DNA, which is much more abundant inside cells. Uno et al. presented a new N-aryl pyrido cyanine derivative, **PC1** (**23**, Scheme 3), that shows a dose-dependent mtDNA staining ability [99]. They demonstrated that this probe has a relatively long absorption wavelength and can be applied to the imaging of mtDNA in living cells with the separation of photons via a lifetime tuning method in stimulated emission depletion nanoscopy (SPLIT-STED). Tan’s group reported a probe called **MitoISCH** (**24**, Scheme 3) using TPP as the guider moiety and specifically responded to the GQ structure in mtDNA [100]. They further indicated that the GQ in mtDNA would affect the energy metabolism mode inside cells.

The microenvironment is also important for the activity of mitochondria, since most of the enzymes are influenced by pH, temperature, and even the viscosity of the surroundings [101]. Using a piperazine-linked naphthalimide as the pH sensor moiety and TPP as the guider, Lee et al. synthesized a probe (**25**, Scheme 3) for the real-time monitoring of pH change in mitochondria in living cells [102]. They observed the process of mitophagy in nutrient-starved cells by using the designed probe. In 2015, Chang’s group introduced a probe, **Mito thermal yellow** (**26**, Scheme 3), with which to detect the temperature of mitochondria [103]. This probe is a derivative of rosamine with rotatable substituents, demonstrating a proportional fluorescence intensity change according to the change in temperature. Further, scientists found that the temperature of mitochondria can be as high as 50 °C, meaning that some mitochondrial recognition events should be re-evaluated [20]. Xiao’s group introduced a rhodamine-B-based probe to monitor the temperature of mitochondria [104]. This probe, termed **Mito-TEM** (**27**, Scheme 3), could anchor on the IMM and thus avoid the signal interference caused by the dispersion due to the dynamics of mitochondria. At present, a 2.0 version of **Mito-TEM** has been presented by the same group and applied to mitochondrial temperature tracking in vivo in a zebrafish model [105]. Chen et al. designed a probe, **NIR-V** (**28**, Scheme 3), by linking 2,3,3-trimethyl-3H-indolenine and 4-(dimethylamino) cinnamaldehyde [106]. This probe was applied to monitor the viscosity changes in the mitochondria in cells as well as in the organs in a mouse model. They demonstrated that **NIR-V** could be used for the detection of viscosity in different organs in diabetic mice models with insulin treatment, thus providing a new tool for the diagnosis of viscosity-related diseases. Zhang et al. introduced a multifunctional mitochondrial-specific probe that can detect SO_2_ and viscosity simultaneously [107]. They constructed the **Mito-MG** (**29**, Scheme 3) probe by combining benzopyrylium salt and 4-dimethylaminobenzaldehyde. This probe was also applied to imaging in both living cells and in vivo. A summary of the properties of the mitochondria-targeted molecular probes is listed in Table 2.

### 2.3. Endoplasmic-Reticulum-Targeted Molecular Probes

#### 2.3.1. Properties of the Endoplasmic Reticulum

The endoplasmic reticulum (ER) is the center of synthesis and transport for most proteins and lipids inside cells [108]. Besides the key role of biomolecule synthesis, the ER also acts as a regulator of many bioprocesses. The ER is the main storage of intracellular calcium, thus regulating the intracellular Ca^2+^ signal transfer [109]. ER also regulates the metabolism of proteins and lipids; moreover, the behavior of ER is also closely linked to the activities of cells and the physiological state of life [7]. ER stress is a series of reactions to restore protein homeostasis activated by misfolded or unfolded protein aggregation as well as calcium ion imbalance in the ER lumen [110], related to many diseases such as cancer [111], diabetes [112], and heart diseases [113]. In recent years, ER-phagy has also been shown to be involved in maintaining ER homeostasis and greatly influence ER as well as cell function [114,115].

Proteins are synthesized in the rough ER, whereas lipids are synthesized in the smooth ER [116]. The two categories of the ER are effectively distinguished by their surface morphologies: The rough ER is rich with ribosomes on the surface and thus appears coarse under electron microscopy. The ER is relatively rich in zwitterionic lipids. Thus, amphipathic, lipophilic cations with moderately sized conjugated systems were proven to be suitable for ER probe designs [117]. The cytosolic face of ER membranes is abundant in ATP-sensitive potassium channel sulfonylurea receptors, whereby ER-targeting probes are often designed and synthesized based on molecules with sulfonamide groups [118].

#### 2.3.2. ER-Targeted Probe Design

**Scheme 4:**
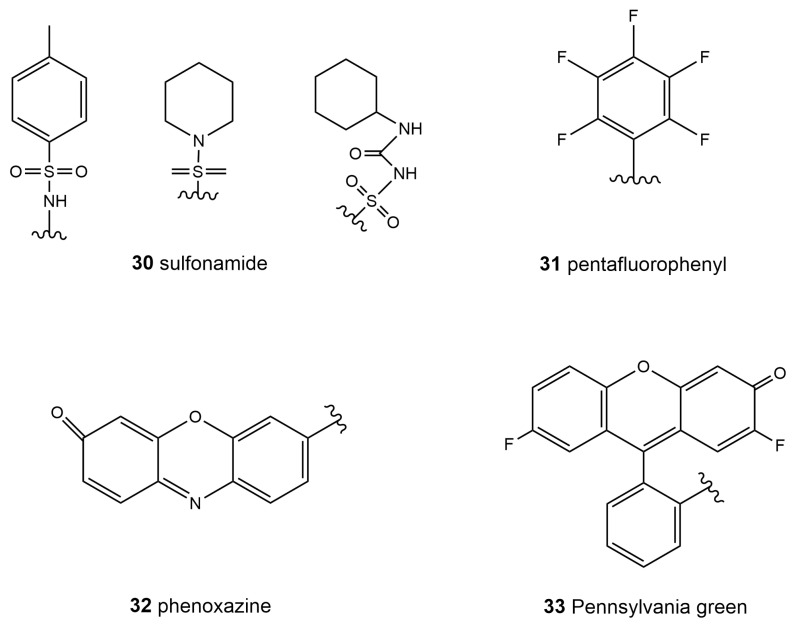
Chemical structures of typical ER building blocks.

Special ATP-dependent K+ channels are abundant on the ER membrane; sulfonylurea and sulfonamide groups can be recognized by the special receptors in these channels [119]. Therefore, molecular probes based on sulfonamide (**30**, Scheme 4) showed great potential for ER specificity. Although commercial probes, such as ER-Tracker Bluewhite DPX, based on the sulfonamide group are now available [16], it is noteworthy that sulfonamide receptors, such as glibenclamide and its derivatives, are still expensive. At this point, scientists have found more affordable molecules with which to design probes that target the ER. For example, pentafluorophenyl (**31**, Scheme 4) is also a general group in probe design for ER targeting. The ER surface proteins that contain the sulfydryl group may react with the pentafluorophenyl group through a halogen-substituted reaction. Moreover, resorufin (**32**, Scheme 4), Pennsylvania green (**33**, Scheme 4), and other groups are also used to design ER-targeting probes [120]. Recently reported hydrophobic resorufamine derivatives, which possess a structural similarity to fluorinated hydrophobic rhodol fluorophores, may accumulate in the ER of mammalian cells and were developed to target the ER [121].

**Scheme 5:**
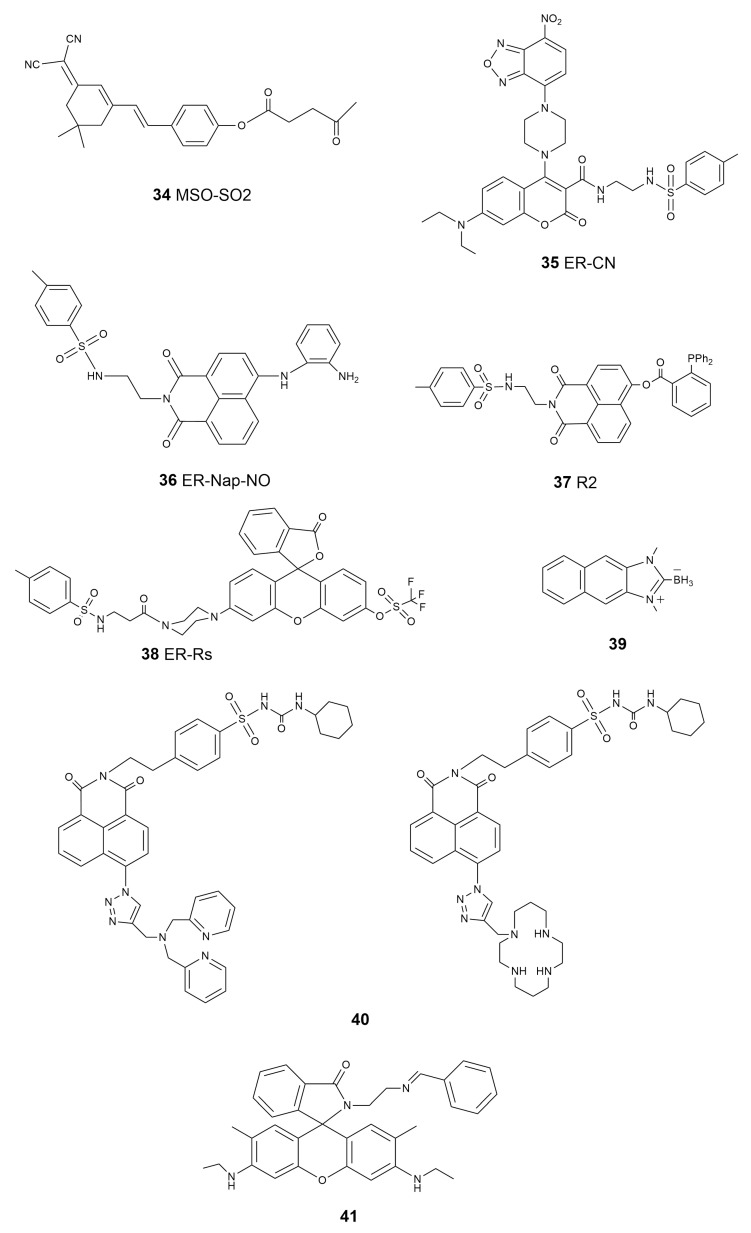
Chemical structure of the ER-targeted fluorescent molecular probes.

As in mitochondria, reactive species, including ROS, RNS, and RSS, are critical factors in the ER for signal transduction and bioprocess regulation [122,123]. Reactive-species-induced ER stress and its association with ER-related diseases have drawn significant attention and concerns. Following the discovery that SO_2_ has the functionality to inhibit ER stress [124], Yue et al. designed a probe, **MSO-SO_2_** (**34**, Scheme 5), with which to detect SO_2_ produced during ER stress induced by dithiothreitol (DTT) [125]. Due to the good ER-targeting ability of dimalononitrile isophorone derivatives, the authors selected levulinic acid as the specific recognition site for SO_2_, as well as a weak electron withdrawing group, and constructed an ER-targeted NIR fluorescent probe based on the dicyanoisophorone framework, realizing directly the visualization of the production as well as consumption of SO_2_ during DTT-induced ER stress. Zhang et al. reported a novel ER-targetable fluorescent probe, **ER-CN** (**35**, Scheme 5), for the visualization of H_2_S in living cells containing piperazine coumarin as the fluorescence platform, a 7-nitro-1,2,3-benzoxadiazole (NBD) moiety as the H_2_S-specific recognition site, and a methyl sulfinamide group as the ER-targeting part [126]. Although the mechanism of NO inducing ER stress has not been fully studied, it has become a fact that NO can alter ER functions and then activate ER-stress-mediated apoptosis [127]. Li et al. designed and synthesized an **ER-Nap-NO** (**36**, Scheme 5) probe composed of naphthalimide as the two-photon fluorophore, o-phenylenediamine as the NO recognition group, and methyl sulfonamide as the ER-targetable group [128]. This new probe was used for the two-photon imaging of exogenous and endogenous NO in the ER of living cells, exhibiting the advantages of a fast response, high selectivity, and low cytotoxicity. Meanwhile, **ER-Nap-NO** showed its capability to monitor NO level in a tunicamycin-induced ER stress system, suggesting that this probe is a promising tool for the function investigation of NO in ER-associated vascular diseases. Linking methyl sulphonamide, as an ER-targeting group, to the 1,8-naphthalimide fluorophore, a probe with which to accurately image exogenous and endogenous HNO in ER was obtained recently by Ye’s group [129]. This new probe, **R2** (**37**, Scheme 5), showed high selectivity, a fast response, a low detection limit, and good biocompatibility in ER-specific cell imaging. Lu et al. constructed a probe that employed triflate as the response site for O_2_•^−^ and p-methylsulfonamide as the ER-specific moiety (**ER-Rs, 38**, Scheme 5) for the imaging of endogenously produced O_2_•^−^ in the ER of living cells [130]. They also demonstrated that this probe, **ER-Rs,** could be applied for O_2_•^−^ monitoring in vivo in tissues and zebrafish models. In 2018, Pak et al. reported a naphthoimidazolium borane probe that could trace the exo/endogenous HClO in the ER of living cells and tissues (**39**, Scheme 5) [131].

In addition to calcium, the ER is also the stockroom of zinc, which is necessary for the proper functioning of living organisms [132]. By introducing a simple sulfonylurea-targeting group, Fang et al. developed probes that realized the remarkable ability to image mobile Zn^2+^ in the ER in different cell lines (**40**, Scheme 5) [133]. Since the discovery of ferroptosis a decade ago, the level of iron in the ER has become a significant marker. The Fenton reaction, which generates Fe^3+^ and highly oxidative hydroxyl radicals from H_2_O_2_ and Fe^2+^ reactions, plays a key role in lipid peroxidation inside cells and initiates the process of ferroptosis [22]. Lee et al. prepared a series of probes through a chemical reaction of rhodamine-ethylene diamine and benzaldehyde to detect Fe^3+^ in the ER [134]. They demonstrated that this particular probe (**41**, Scheme 5) showed good selectivity to the ER-localized Fe^3+^ in HepG2 cells. A summary of the properties of the ER-targeted molecular probes is given in Table 3.

### 2.4. Lysosome-Targeted Molecular Probe

#### 2.4.1. Properties of Lysosomes

Lysosomes are spherical catabolic organelles that contain more than 50 kinds of hydrolases, such as protease, phosphatase, lipase, and nuclease, which are used to metabolize and decompose a variety of endogenous and exogenous macromolecular substances, such as sugar, lipids, glycolipids, glycosaminoglycans, nucleic acids, and proteins [135]. As one of the most important digestive organelles in a cell, lysosomes have been proven to be involved in controlling the recycling and signal transduction processes of cell growth, division, differentiation, and cell death; they are vital elements in maintaining metabolic homeostasis, intracellular signal transduction, and plasma membrane repair as well as secretion [136]. Numerous studies have revealed that the dysfunction of lysosomes is related to various diseases, including cancers, atherosclerosis, and neurodegenerative disorders [137]. In the most recent research, a novel therapeutical strategy, termed lysosome-targeting chimaera (LysoTAC), has emerged for disease treatments that rely upon the digestion function of lysosomes [138,139]. Therefore, the imaging of lysosomes and the monitoring of their activities are attractive to people in a wide range of research fields, such as biology, pathology, pharmacology, and biochemistry.

As the activity of hydrolase is usually the highest under acidic conditions (pH = 4.5~5), lysosomes continuously pump protons into the lysosome through V-type ATPase, i.e., a proton pump, to maintain a stable acidic microenvironment and metabolic capacity [140]. The acidic pH in the lysosome chamber is also an important characteristic, mainly regulating the circulation of proteins and macromolecules. Because of this lower pH of the lysosome chamber compared to other parts of the cell, the pH-sensitive probe has attracted the attention of scientists when designing lysosome-targeted probes.

#### 2.4.2. Lysosome-Targeted Probe Design

Lysosome-targeted molecular probes are usually derived from lipophilic amines, a lipophilic weak base group. Amine-containing molecules can accumulate in lysosomes through passive diffusion or active transport actions, including endocytosis and autophagy; however, compared to the accumulation mechanism mediated by passive diffusion, endocytosis or autophagy contributes less to the number of accumulation probes [141,142]. The majority of probe design is still focused on the accumulation mechanism mediated by passive diffusion. Small molecules below 1 kDa diffuse freely in the cell membrane system [143]. Once the small-molecule probe diffuses into the lysosome, the amine moiety can be protonated. Due to the impermeability of the membrane, a probe that becomes positively charged will be trapped in the lysosome organelle. For example, commercial LysoTracker probes, constructed based on the same lipophilic amino moiety, have been widely applied to label lysosomes with different fluorescences [144,145].

In addition to weakly alkaline anchoring, scientists have also reported fluorescent probes based on an “AND” logic gate strategy. Probes that only release fluorescence signals in an acidic environment and after reacting with analytes are designed for the detection of lysosomal location matters. These kinds of molecules may not accumulate in lysosomes automatically, but would still exhibit an excellent signal-to-noise ratio for lysosome-targeted cell imaging.

**Scheme 6:**
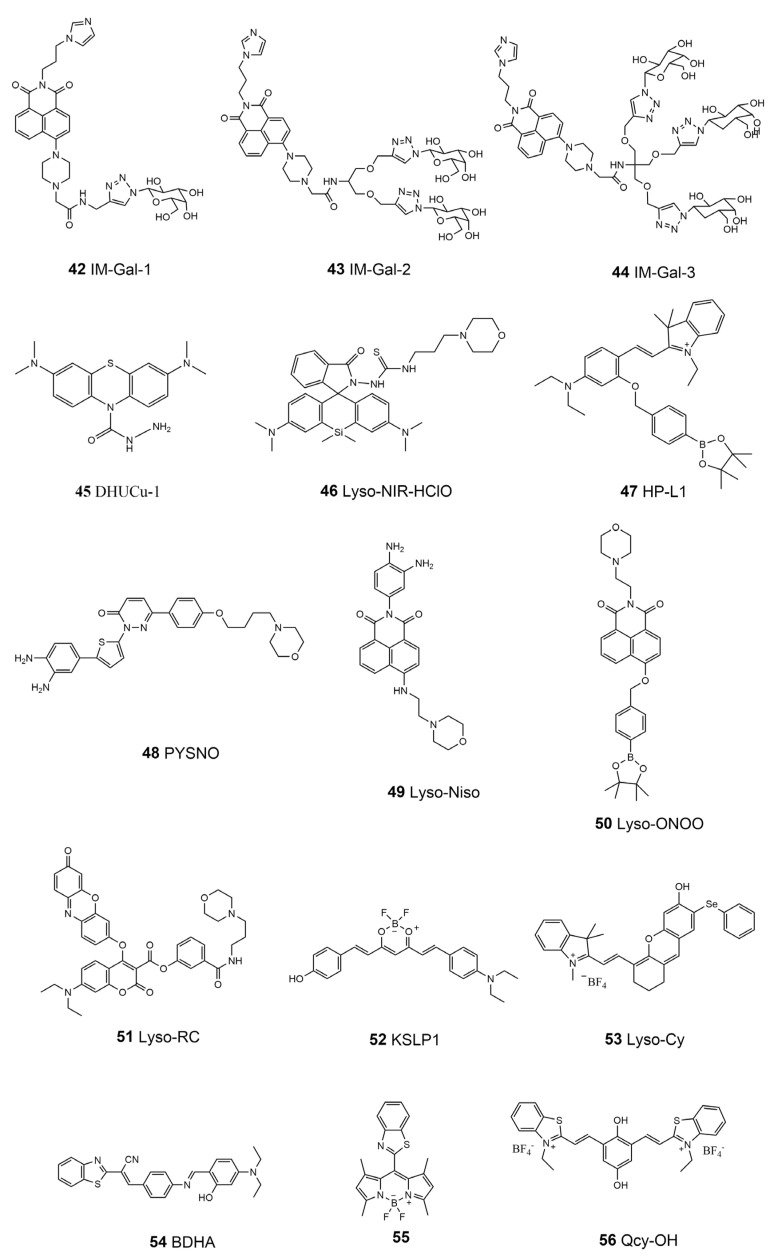
Chemical structure of the lysosome-targeted fluorescent molecular probes.

Metal ions are involved in the mediation of the activation of lysosomal enzymes, including the proton pump V-type ATPase [140]. On the other hand, lysosomes are the main regulators of the metabolism of ions such as iron [146,147]. The liver is the main organ for storing iron in the body. The concentration of iron ions (especially Fe^3+^) in the lysosome of liver cells is one of the important biological indicators for diagnosing liver diseases such as cirrhosis, liver injury, and liver cancer [148]. Wang’s group designed three new Fe^3+^ fluorescence probes, using galactose as the liver-cell-targeting group, imidazole as the lysosome-targeting group, and naphthimide, with good photophysical and chemical properties, as the fluorescence response group to obtain monodentate, bidentate, and tridentate galactose probes—**IM-Gal-1**, **IM-Gal-2**, and **IM-Gal-3** (**42–44**, Scheme 6) [149]. A fluorescence enhancement signal is generated in the presence of Fe^3+^ due to photoinduced electron transfer. Galactosyl probes can specifically recognize the overexpressed asialoglycoprotein receptor (ASGPR) in hepatocytes, which enables the probes to exhibit good liver-targeting ability. Cu^2+^ in lysosomes is reported to relate to Munk’s syndrome, Wilson’s disease, Alzheimer’s disease, and other diseases [150]. Chen et al. designed and synthesized a lysosome-targeted fluorescence probe, **DHUCu-1** (**45**, Scheme 6), with near-infrared emission using methylene blue as the fluorescence carrier [151]. It can detect and image Cu^2+^ in lysosomes, in addition to having the advantages of good water solubility and low toxicity. When the nitrogen-containing structural probe coordinates with Cu^2+^ in lysosomes, a conjugated system is formed to generate a fluorescence-enhanced signal.

Unsurprisingly, the reactive species are also important in lysosomes. For example, ROS and RNS may induce lysosomal oxidative stress; the abnormal homocysteine (HCy) level may cause or aggravate cardiovascular and Alzheimer’s disease [152,153]. Mao et al. reported a lysosome-targeted near-infrared fluorescence probe, **Lyso-NIR-HClO** (**46**, Scheme 6), for highly selective as well as sensitive detection and imaging of endogenous and exogenous HClO in lysosomes [154]. This probe is composed based on Si-rhodamine modified with a morpholine unit as a lysosome-targeting group and a HClO-mediated cyclization reaction site as a response group. This probe was applied for HClO imaging in living cells and mice models. In 2016, Liu et al. introduced a probe (**HP-L1**, **47** Scheme 6) activated in an acidic environment for detecting H_2_O_2_ in lysosomes [155]. The fluorescence probe uses borate as the response sensing unit of H_2_O_2_ and spirobenzopyran as the pH-switchable fluorophores. The probe is constructed based on the “AND” logic gate method, meaning that it can only emit strong fluorescence if it reacts with H_2_O_2_ in acidic conditions in the lysosome. Recently, Zhou et al. designed a lysosomal-targeted NO fluorescence probe, **PYSNO** (**48**, Scheme 6), with high sensitivity and selectivity on the basis of a pyridazinone scaffold and photo-induced electron transfer (PET) mechanism, using morpholine as the acidic lysosomal-targeting group [156]. The fluorescence was turned on through the photo-induced electron transfer mechanism, which was used to explore the relationship between myocardial NO production and myocardial fibrosis in mice models. Based on the principle of PET, Yu et al. introduced the lysosome-targeted N-aminoethyl morpholine into the efficient two-photon fluorescence carrier naphthalimide. Using o-phenylenediamine as the recognition group, they designed a lysosome-targeted two-photon fluorescent free probe, **Lyso-Nino** (**49**, Scheme 6), which is highly selective and sensitive to NO [157]. The probe was used to capture NO from the lysosomes of macrophages for the first time and successfully monitored the production of NO in lysosomes under different stimuli. In the most recent study, Wang et al. synthesized a lysosomal-targeted naphthalimide-derived fluorescent probe, **Lyso-ONOO** (**50**, Scheme 6), that can monitor the exogenous and endogenous peroxynitrite (ONOO−) of human hepatic stellate cells [158]. The probe uses the phenyl borate pinacol ester group as the recognition group of ONOO− and morpholine as the lysosomal-targeting group. This probe can infer the order of severity in liver injury processes through the change in the level of ONOO−. In 2018, Zhang et al. developed a lysosome-targeted fluorescence probe, **Lyso-RC** (**51**, Scheme 6), for the simultaneous detection of Cys/Hcy, GSH, and H_2_S [159]. The target group of lysosomes is the morpholine group, and the probe contains two fluorophores, resorufin and 7-diethylaminocoumarin. This particular probe exhibits different fluorescences when responding to different RSS. Its emission spectra are generally free of crosstalk, which allows for simultaneous detection.

The lysosomal microenvironment affects the molecular transportation and activity of the enzymes, thus being related to the lysosomal function and status [160]. Li and co-workers proposed a curcumin-based polar near-infrared lysosomal-targeting fluorescence probe, **KSLP1** (**52**, Scheme 6), with which to monitor the changes in lysosomal polarity in the process of cell aging [161]. Using this probe, scientists found that the polarity of lysosomes in aging cells increased for the first time and proposed the potential mechanism of changes in lysosomal polarity in the process of cell aging. The viscosity of lysosomes plays an important role in biological regulation [162]. The real-time tracking and monitoring of the dynamic changes in lysosomal viscosity are of great significance for diagnosing viscosity-related lysosomal diseases. Recently, Zeng’s group constructed a near-infrared lysosome-targeted viscosity probe, **Lyso-cy** (**53**, Scheme 6), with which to monitor the change in viscosity of biological systems [163]. The phenyl selenide group was introduced into the oxaanthrene indole dye, making the probe an ideal lysosome-targeting tracer. In a viscous medium, this probe has strong fluorescence emission near 710 nm and has been demonstrated to work well in the monitoring of the change in the lysosomal viscosity of living cells. The benzothiazole group was found to target lysosomes in living cells. Chen et al. synthesized a lysosome-targeted fluorescent probe, **BDHA** (**54**, Scheme 6), based on benzothiazole compounds, realizing the dual detection of OCl- and viscosity [164]. BDHA can be used to image the viscosity and OCl- level of HeLa cells and zebrafish. In 2022, Shi et al. designed and synthesized a BODIPY-based fluorescence probe (**55**, Scheme 6) with a rotatable meso-benzothiazole group, showing both good viscosity responsiveness and AIE properties [165]. This probe can sense lysosomal viscosity changes induced by lipopolysaccharides, nystatin, a low temperature, and dexamethasone in living cells, which can be further applied in autophagy monitoring via tracking viscosity changes. Recently, a new NIR fluorescent probe, **Qcy-OH** (**56**, Scheme 6), was developed by Zeng’s group to track lysosomal pH changes [166]. Two benzothiazolium units were introduced in the backbone of the probe to enhance the selectivity for intracellular lysosomes. This sulfur-driving lysosome-targeting ability of the probe affords a guarantee for achieving the long-term monitoring of lysosomal pH biology through the elimination of harmful protonating effects of the probe. A summary of the properties of the lysosome-targeted molecular probes is listed in Table 4.

### 2.5. Dual-Targeted Molecular Probe

Different organelles play their roles inside cells as parts; however, as a whole, they must communicate with each other to keep entities running smoothly. Different molecules, such as ions, lipids, and proteins, transfer between different organelles under sophisticated regulation. For example, the unfolded protein response (UPR) of the ER and mitochondria may cause the translocation of some transcription factors toward the nucleus to initiate feedback bioprocesses [167,168]. The fission of mitochondria requires contact between mitochondria and the ER [169,170]. Lysosomes may attend to the autophagy of different organelles, such as mitophagy, through signal-induced recruitment [171]. Therefore, dual-targeted probes that can monitor different organelles simultaneously have attracted more and more interest because they have shown great potential for the investigation of communication and signal transfer processes between different organelles.

**Scheme 7:**
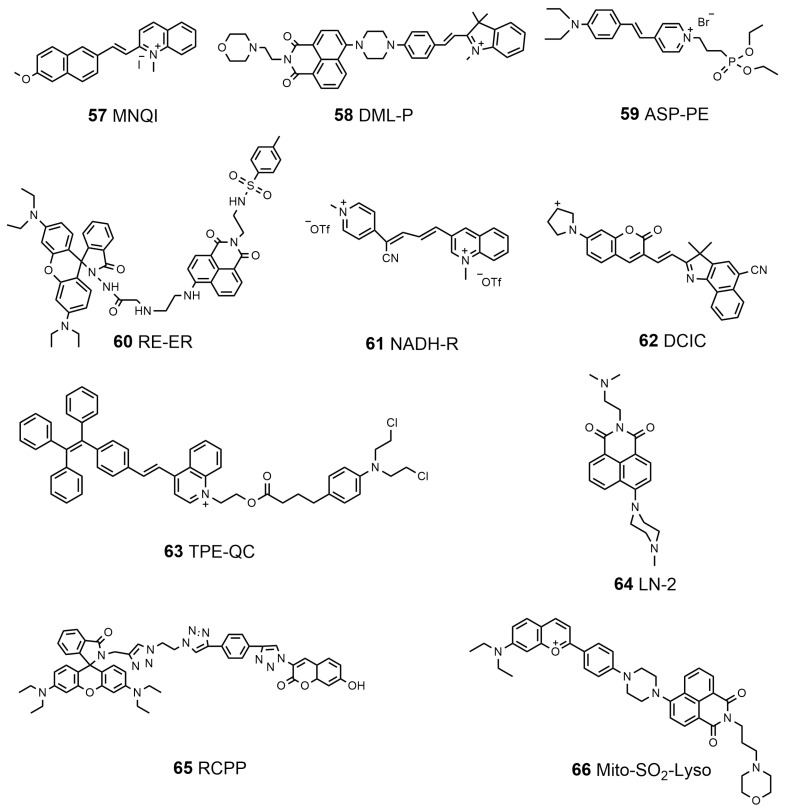
Chemical structure of the dual-targeted molecular probes.

In recent years, many functional multi-organelle-targeting probes have been developed. Lin’s group designed and synthesized a fluorescent probe, **MNQI** (**57**, Scheme 7), which has a weak electronic donor and a positively charged group to bind DNA, for the visualization of the mitochondria and nucleus in dual channels [172]. Furthermore, they proved that the probe could reversibly detect the changes in MMP levels from both the localization and the emission color; reversible changes in cell viability have been successfully observed with the probe. The same group employed a semi-cyanine unit as the SO_2_-responsive site, a naphthalimide unit and semi-cyanine unit as the fluorophores, and semi-cyanine as well as morpholine groups to target mitochondria and lysosomes, constructing the first dual-targeting organelle-targeted fluorescent probe, **DML-P** (**58**, Scheme 7), capable of tracing the mitochondria and lysosome SO_2_ simultaneously [173]. Most recently, Ho et al. utilized an electron push–pull π-conjugation skeleton and a rarely reported diethyl phosphonate moiety on the pyridinium ring to develop an AIE-based bioprobe, **ASP-PE** (**59**, Scheme 7), for dual plasma membrane–mitochondria targeting through its phosphonate moiety and pyridinium, visualizing the changes in the membrane tension of the plasma membrane as well as mitochondria in response to varied osmotic pressure and substrate stiffness [174]. Therefore, this probe revealed the significance of actin and microtubules for plasma membranes and mitochondria in response to mechanobiological stimulations. A probe named **RT-ER** (**60**, Scheme 7), which could image the ER and lysosomes via sequencing under the stimulation of ClO-, was designed by Shan et al. through combining rhodamine B and 1,8-naphthalimide [175]. It was proven that this probe showed fast responses and was highly specific to ClO-, in addition to having an extremely large linear range, allowing it to be used in quantitative analyses in vitro. Duan et al. rationally designed a fluorescent probe, **NADH-R** (**61**, Scheme 7), via a simple graft of pyridiniumylbutenenitrile on a 1-methylquinolinium moiety in the 3-position, which could visualize exogenous and endogenous NAD(P)H generation in living cells. People applied NADH-R to monitor the fluctuation in the level of NAD(P)H under metabolic perturbation, and observed decreased NAD(P)H levels in the brains of stroke mice models. These results indicated that the new probe has a certain reference value for the treatment of clinical diseases [176]. Based on the TICT mechanism, a new NIR pH-dependent fluorescent probe, **DCIC** (**62**, Scheme 7), with a push–pull electronic moiety was synthesized by Wang et al. to identify the lysosome viscosity. The fluorescence quenching of **DCIC** did not occur with the environment changing from acidic to neutral or alkaline, providing a basis for multi-organelle localization [177]. **DCIC** can monitor lysosomal viscosity fluctuations while localized towards lysosomes, mitochondria, the Golgi apparatus, and the ER. Recently, Zhuang et al. developed an esterase-responsive prodrug, **TPE-QC** (**63**, Scheme 7), for dual organelle-targeted imaging and synergetic chemo-photodynamic cancer therapy [178]. They demonstrated that activated **TPE-QC** accumulated in both lysosomes and mitochondria. Benefitting from the overexpressed esterase in cancer cells, **TPE-QC** could be efficiently activated in cancer cells rather than in normal cells, resulting in enhanced anticancer potency. Lin’s group synthesized a fluorescent probe, **LN-2** (**64**, Scheme 7), to visualize cell death based on its subcellular immigration from lysosomes towards the nucleus by regulating the binding affinity of the probe to lysosomes and DNA [179]. LN-2 was successfully used to display cell death induced by hydrogen peroxide and apoptosis induced by rotenone. It is a potential tool for studying apoptosis-related bioprocesses. Yapici et al. designed and synthesized a broad-range pH probe, **RCPP** (**65**, Scheme 7), to detect organellar acidity/alkalinity by integrating an acidic-pH-responsive rhodamine label with an alkaline-pH-responsive coumarin moiety [180]. Depending on the organellar acidity/alkalinity, **RCPP** can simultaneously move between mitochondria and lysosome subcellular locations, facilitating the simultaneous monitoring of acidity/alkalinity alterations in mitochondria and lysosomes in living cells. Recently, Yin’s group designed a dual-targeting SO_2_ fluorescent probe, **Mito-SO_2_-Lyso** (**66**, Scheme 7), based on the FRET regulation strategy, which realized the ratio detection of endogenous and exogenous SO_2_ in vivo [181]. Among them, benzopyranium salt units and morpholine groups were conjugated to target mitochondria and lysosomes. They demonstrated that the **Mito-SO2-Lyso** could quantitatively detect sulfite concentration in Yuba and crystal sugar, showing potential applications in cell imaging and food analyses. A summary of the properties of the dual-targeted molecular probes is listed in Table 5.

## 3. Summary and Outlook

The development of fluorescent molecular probes provides powerful tools in the fields of cell-related research. At this point, elaborate cell images covering subcellular information, such as the patterns and statuses of organelles, can be obtained by utilizing specially designed probes. From these images, it is possible to investigate the relationship between particular organelles and different bioprocesses, revealing the signal molecules that play a key role in regulating bioactivity, explaining the molecular mechanisms of some diseases and bringing potential therapeutic targets to light. Hence, advanced fluorescent molecular probes facilitate the understanding of biology, pathology, and pharmacology. Here, we review the probes developed in the past decade for targeting organelles, such as nucleus, mitochondria, ERs, and lysosomes. We mostly focused on the probes proposed in the past five years and discussed the types of building blocks that can be selected for achieving organelle specificity according to the unique chemical/physical properties of different organelles. We also introduced the representative probes that can respond to special intracellular chemical/biological reagents at the subcellular level. The novel probes certainly showed advanced performances in comprehensive applications, but some drawbacks and challenges remain. We believe some of the following directions might receive more attention in the future in order to promote the capacity of the probes.

(1) Low cytotoxicity: The cytotoxicity of the fluorescent molecular probes includes two parts. The first is the phototoxicity generated when the probes were excited by irradiation sources. Under the excitation conditions during cell imaging, the generation of ROS is inevitable. The exceedingly exogenous ROS may break the intracellular redox balance and cause oxidative damage to intracellular biomacromolecules, including DNA, proteins, and lipids, thus influencing the statuses of organelles and living cells. This property of the molecules has been applied to photodynamic therapy for disease treatment; however, it is a limitation of probes in the application of cell imaging, especially in cases of super-resolution and longtime imaging, which need higher emission energy. The modification of the fluorophore is still necessary to obtain a probe that works better inside cells. Chen and co-workers have introduced a triplet-state engineering strategy with which to construct mitochondria-targeted probes with reduced phototoxicity, providing a potential strategy for this direction [182]. The other part of the cytotoxicity of the molecular probes is the interruption of cell activity from the molecules themselves. For example, the membrane potential of the mitochondria will be somehow neutralized by the cationic molecules accumulated around the IMM. The underlying mechanism can introduce interferences with the normal activity of the respiratory chain reaction and thus cause the dysfunction of mitochondria. Therefore, a suitable targeted strategy is needed. The mitochondria-penetrating peptides (MPPs) are promising choices [183,184], which were reported to guide exogenous molecules into the mitochondrial location. Similarly, the sulfonamide group that can react with the K^+^ channel on the ER surface may influence the bioactivity of the ER. Therefore, several ER localization signal sequences that guide intracellular protein distribution, such as ER retention signal sequences, KDEL (—Lys-Asp-Glu-Leu-COOH), and ER insertion signal sequences, Eriss (ER insertion signal sequence), have been conjugated with molecular probes for ER-targeted imaging [185]. Some special short peptides, named nuclear localization signals (NLSs), or other chemical motifs that may recognize the importins in nuclei were applied to construct the nucleus-targeted probes [186].
(2) The capacity of in vivo imaging: Fluorescent imaging in vivo is always challenging but engaging because it provides the most straightforward biological information. Many of the probes developed at this point possess a relatively short wavelength of excitation/emission located in the UV–Vis wavelength region. This region overlaps with the excitation/emission spectra of biomolecules and biosystems. Therefore, the result might be cluttered by the high background signals. In addition, the absorbance of the short-wavelength light by the biological samples will also limit the application of the probes in deep sample penetration imaging. The satisfying wavelength for in vivo imaging is in the near-infrared range (NIR, 650–950 nm), which can avoid interference form the biological samples. A number of NIR fluorophores have been developed, suggesting the importance of considering the link design with particular moieties for in vivo imaging at the organelle level. Another encouraging method is the design of two-photon-excited fluorophores, which can be excited by two lower-energy NIR photons. Therefore, two-photon fluorescent microscopy can be applied for imaging in vivo by using fluorescent probes with an increased penetration depth and other advantages, such as a prolonged observation time.
(3) Biological guidance: Most molecular probes are developed by chemists, but the utilization of probes is most likely carried out by biologists. The gap between these two fields is obvious, implying that communication between scientists is integral to major breakthroughs and beyond. Our standpoint suggests that the development of biology should guide probe design. The studies on ferroptosis created an urgent demand for the detection of cellular iron and lipid peroxidase, as an example [187]. It was recently found that the calcium transients on the ER surface would trigger the process of autophagy [21], meaning that the ER-targeted Ca^2+^ probes are promising tools for autophagy monitoring. Moreover, the development of novel probes may also encourage biologists to discover unknown bioprocesses. For example, high-resolution methods can assist in distinguishing the different modes of mitochondrial fission; this may explain how other organelles participate in modulating the fission process in cells [188]. Furthermore, a series of novel probes that can monitor the membrane tension on the plasma membrane and other organelles were developed recently and promote the understanding of mechanobiological processes, thus indicating the importance of mechanoforce as an interesting parameter in the regulation of bioactivities [189,190,191,192]. Therefore, it is critical for scientists in different fields to exchange their cutting-edge knowledge to discover the most suitable molecular probes, thus advancing the growth of science.

Overall, the journey of organelle-targeted fluorescent molecular probes is just beginning. Deeper growth of chemical and biological knowledge will certainly lead to new probes as powerful tools for the relative fields.

## Figures and Tables

**Table 1:** Properties of the nucleus-targeted fluorescent molecular probes.

Number	Probe	Analyte	λex/nm	λem/nm	Φ	Probe Concentration	LOD	Ref.
1	hoeTMP	DNA	\	\	\	\	\	[35]
2	HoeSR	DNA	532	590	0.09	\	\	[37]
3	\	DNA	530	680	0.32	\	\	[38]
4	\	DNA	455, 492	612, 716	0.31, 0.41	\	\	[40]
5	\	dsDNA	480	577, 567	0.022, 0.015	\	\	[44]
6	\	DNA	477	610	0.42	\	\	[42]
7	BEB-A	Cys	543	616	\	5 μM	27 nM	[46]
8	DAOTA-M2	G-quadruplexes	\	\	\	\	\	[51]
9	TP-2Bz	G-quadruplexes	488	600–670	\	\	\	[53]
10	STDBT	Ca^2+^	488	524–540	\	\	\	[54]
11	NucPE1	H_2_O_2_	514	530	0.626	\	\	[58]
12	Hoe-Rh-NO	NO	405	463\603	\	5 μM	58 nM	[59]

**Table 2:** Properties of the mitochondria-targeted fluorescent molecular probes.

Number	Probe Name	Analyte	λex/nm	λem/nm	Φ	Probe Concentration	LOD	Ref.
17	HQPQ-B	H_2_O_2_	450	575	\	10 μM	1.76 μM	[83]
18	TFP	H_2_O_2_ ATP	710	430–530 550–650	0.31 0.72	10 μM	68 ± 5 nM 33 ± 2 μM	[84]
19	NPClA	SO_2_ ClO^−^	395	482 425	\	10 μM	250 nM 16.6 nM	[85]
20	MitoClO	HClO	480	529	0.44	1 μM	0.52 μM	[86]
21	\	NO	488	500–600	0.11	4 μM	4.8 nM	[95]
22	\	GSH	550	599	0.238	10 μM	109 nM	[96]
23	PC1	mtDNA	532	546	0.09	\	\	[99]
24	MitoISCH	mtDNA G4	560	580–800	\	\	\	[100]
25	\	pH	407	525	\	\	\	[102]
26	Mito thermal yellow	Temperature	\	\	\	\	\	[103]
27	Mito-TEM	Temperature	559	575–620	\	\	\	[104]
28	NIR-V	Viscosity	580	700	\	\	\	[106]
29	Mito-MG	SO_2_ Viscosity	675	675 770–781	\	10 μM	0.24 μM \	[107]

**Table 3:** Properties of the ER-targeted fluorescent molecular probes.

Number	Probe Name	Analyte	λex/nm	λem/nm	Φ	Probe Concentration	LOD	Ref.
34	MSO-SO_2_	SO_2_	560	670–720	\	10 μM	12.3 nM	[126]
35	ER-CN	H_2_S	405	470–510	\	10 μM	4.9 μM	[127]
36	ER-Nap-NO	NO	440	538	0.214	10 μM	3.3 nM	[129]
37	R2	HNO	450	555	\	10 μM	32 nM	[130]
38	ER-Rs	O_2_•^−^	500	558	0.41	5 μM	0.12 μM	[131]
39	\	HClO	326	450	0.29	10 μM	3.6 μM	[132]
40	\	Zn^2+^	346	414, 406	0.25 0.041	1 μM 1 μM	47 pM 0.71 nM	[134]
41	\	Fe^3+^	528	552	\	\	\	[135]

**Table 4:** Properties of the lysosome-targeted fluorescent molecular probes.

Number	Probe Name	Analyte	λex/nm	λem/nm	Φ	Probe Concentration	LOD	Ref.
42	IM-Gal-1	Fe^3+^	405	510–550	\	\	(129.3 ± 0.5) nM	
43	IM-Gal-2				\		(77.35 ± 0.05) nM	[150]
44	IM-Gal-3				\		(132.5 ± 2.3) nM	
45	DHUCu-1	Cu^2+^	620	686	\	10 μM	19.1 nM	[152]
46	Lyso-NIR-HClO	HClO	635	680	\	5 μM	20 nM	[155]
47	HP-L1	H_2_O_2_	520	584	\	5 μM	0.23 μM	[156]
48	PYSNO	NO	405	515–565	0.28	5 μM	242 nM	[157]
49	Lyso-Nino	NO	440	520–560	0.3	5 μM	5 nM	[158]
50	Lyso-ONOO	ONOO^−^	450	555	\	10 μM	0.13 μM	[159]
51	Lyso-RC	Cys/HCy	376	480	\	10 μM	27 nM/33 nM	
		GSH	438	542	\		16 nM	[160]
		H_2_S	580	602	\		0.38 μM	
52	KSLP1	Polarity	560	650–750	\	10 μM	\	[162]
53	Lyso-Cy	Viscosity	635	685–785	0.41	5 μM	\	[164]
54	BDHA	Viscosity	457	626	\	10 μM	\	
		ClO^−^	405	570–620	\		2.8 μM	[165]
55	\	Viscosity	488	520–600	0.329	10 μM	0.38 cP	[166]
56	Qcy-OH	pH	635	650–750	0.187	10 μM	\	[167]

**Table 5:** Properties of the dual-targeted molecular probes.

Number	Probe Name	Targeting Site	Analyte	λex/nm	λem/nm	Φ	Probe Concentration	LOD	Ref.
57	MNQI	Mitochondria Nucleus	DNA	488	570 (aq) 690 (s)	\	\	\	[173]
58	DML-P	Mitochondria Lysosome	SO_2_	405	530	0.89	10 μM	0.82 μM	[174]
59	ASP-PE	Mitochondria Plasma membrane	Membrane tension	515	610	\	\	\	[175]
60	RT-ER	ER Lysosome	ClO^−^	456	576	0.1245	10 μM	3.37 μM	[176]
61	NADH-R	Mitochondria ER	NAD (P)H	610	657	\	5 μM	12 nM	[177]
62	DCIC	Lysosome, mitochondria, ER Golgi apparatus	Viscosity	500	630	0.1969	10 μM	1.0 cp–438.4 cp	[178]
63	TEP-QC	Lysosome, mitochondria	Esterase	488	520–620	0.126	10 μM	\	[179]
64	LN-2	Lysosome Nucleus	Cell death	488	500–550	\	1 μM	\	[180]
65	RCPP	Mitochondria Lysosome	pH	592.5 405	608–648 419–465	\	10 μM	\	[181]
66	Mito-SO_2_-Lyso	Mitochondria Lysosome	SO_3_^2−^ HSO_3_^−^	470	570 650	\	10 μM	0.017 μM 0.3 μM	[182]

## Data Availability

No new data were created or analyzed in this study. Data sharing is not applicable to this article.
